# Research progress on arsenic, arsenic-containing medicinal materials, and arsenic-containing preparations: clinical application, pharmacological effects, and toxicity

**DOI:** 10.3389/fphar.2024.1338725

**Published:** 2024-03-01

**Authors:** Yichu Yang, Yiye Li, Ran Li, Zhang Wang

**Affiliations:** ^1^ College of Pharmacy, Chengdu University of Traditional Chinese Medicine, Chengdu, China; ^2^ State Key Laboratory of Southwestern Chinese Medicine Resources, Chengdu University of Traditional Chinese Medicine, Chengdu, China; ^3^ College of Ethnomedicine, Chengdu University of Traditional Chinese Medicine, Chengdu, China

**Keywords:** arsenic, arsenic-containing medicinal materials, arsenic-containing preparations, clinical application, pharmacology, toxicity

## Abstract

**Introduction:** The toxicity of arsenic is widely recognized globally, mainly harming human health by polluting water, soil, and food. However, its formulations can also be used for the clinical treatment of diseases such as leukemia and tumors. Arsenic has been used as a drug in China for over 2,400 years, with examples such as the arsenic-containing drug realgar mentioned in Shennong’s Herbal Classic. We have reviewed references on arsenic over the past thirty years and found that research has mainly focused on clinical, pharmacological, and toxicological aspects.

**Results and Discussion:** The finding showed that in clinical practice, arsenic trioxide is mainly used in combination with all-trans retinoic acid (ATRA) at a dose of 10 mg/d for the treatment of acute promyelocytic leukemia (APL); realgar can be used to treat acute promyelocytic leukemia, myelodysplastic syndrome, and lymphoma. In terms of pharmacology, arsenic mainly exerts anti-tumor effects. The dosage range of the action is 0.01–80 μmol/L, and the concentration of arsenic in most studies does not exceed 20 μmol/L. The pharmacological effects of realgar include antiviral activity, inhibition of overactivated lactate dehydrogenase, and resistance to malaria parasites. In terms of toxicity, arsenic is toxic to multiple systems in a dose-dependent manner. For example, 5 μmol/L sodium arsenite can induce liver oxidative damage and promote the expression of pro-inflammatory factors, and 15 μmol/L sodium arsenite induces myocardial injury; when the concentration is higher, it is more likely to cause toxic damage.

## Graphical Abstract

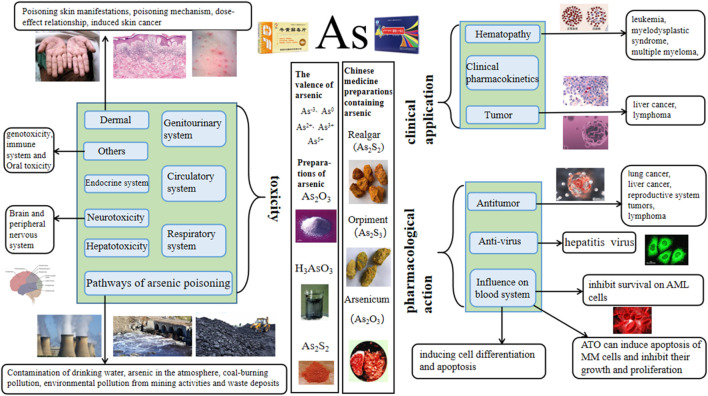

## 1 Introduction

Arsenic, commonly known as pi, is located in the fourth cycle and the VA group in the periodic table of elements. Its atomic number is 33, and the element symbol is As. It is a toxic metalloid element. It has an atomic weight of 74.92159. It is located between phosphorus and antimony, and its physical and chemical properties are similar to those of phosphorus. It can form alloys and is easy to covalently combine with carbon, hydrogen, and oxygen. Arsenic exists in inorganic form, organic form, and different oxidation states. It is widely distributed in nature. It not only exists in the crust, groundwater, soil, seawater, river water, atmosphere, rock strata, and coal seams (Wu, 2002; Jessica, 2020) but also in air and food at low levels. Arsenic mainly exists in the form of sulfide in nature, accompanied by a small amount of natural arsenic and metal arsenide (Pan et al., 2013). Arsenic has many different valence states. The arsenic element present in nature mainly has five valence states: As^3−^, As^0^, As^2+^, As^3+^, and As^5+^. Arsenic in different valence states has varying levels of toxicity. In general, inorganic arsenic is more toxic than organic arsenic, and trivalent arsenic is more toxic than pentavalent arsenic (Zhang et al., 2021a). The toxicity mechanisms of inorganic arsenic include inducing oxidative damage (Kitchin et al., 2010), activating mitosis and inducing genetic toxicity damage, interfering with methyl transfer to DNA (Kundu et al., 2011), and perturbing histones (Chervona et al., 2012).

The toxicity of arsenic has always been a concern, and it is considered a first-class carcinogen in humans. In recent years, a large number of epidemiological and toxicological studies have proven that it is directly related to the incidence of skin cancer, bladder cancer, kidney cancer, lung cancer, and gastric cancer (Tchounwou et al., 2004; Palma-Lara et al., 2020). Individuals highly exposed to arsenic may experience acute, subacute, or chronic poisoning symptoms, which are characterized by skin damage, cardiovascular symptoms, and multiple organ failure (Nurchi et al., 2020). In addition, it can cause reproductive and developmental toxicity, neurotoxicity, and immunotoxicity and induce various cancers in humans. Inorganic arsenic compounds are also carcinogenic to experimental animals. Arsenic exposure is positively correlated with the incidence rate of human kidney cancer, liver cancer, and prostate cancer (Xu et al., 2014). However, the clear mechanism of arsenic carcinogenesis has not yet been confirmed. Possible mechanisms include changes in DNA repair, DNA methylation oxidative stress, and genotoxicity, (Kuivenhoven et al., 2023).

Among the various forms of arsenic, ATO (As_2_O_3_) is the most widely used. As_2_O_3_ is a kind of slightly acidic amphoteric oxide, which is a white frost-like powder or crystal that is slightly soluble in water to generate arsenous acid. It is soluble in acid and alkali, and its amorphous solid is insoluble in ethanol. As_2_O_3_ has a relative molecular weight of 197.84, a melting point of 315°C, a boiling point of 457.2°C, and a saturated vapor pressure of 13.33 kPa (Hao et al., 2020). It is easy to sublimate when heated. The severe toxicity and narrow treatment window of As_2_O_3_ limit its frequency and scope of clinical use. However, when made into appropriate formulations and administered correctly, it has unique effects on various diseases such as asthma, syphilis, and ulcers, as well as difficult conditions such as leukemia and malignant tumors. As_2_O_3_ has been used as a drug for more than 2000 years (Rao et al., 2013). As early as the 1930s, Western scholars applied it to treat leukemia, but the results were unsatisfactory. Until the 1970s, Chinese scholars developed ‘Ailing Yihao’ with arsenic as the main component, which was applied to a variety of blood diseases. It exerts remarkable effects on acute promyelocytic leukemia (APL) (Chen et al., 2011), and arsenic has dual pharmacological effects of inducing differentiation and apoptosis of leukemia cells (Zhu et al., 2012). The therapeutic effect of As_2_O_3_ is time-dependent and dose-dependent. A small amount of As_2_O_3_ does not work for a short time, but a large amount of As_2_O_3_ can easily cause toxic side effects. Therefore, maintaining a stable and effective blood arsenic concentration for a long time and increasing and reducing toxicity on this basis (Sönksen et al., 2022) are urgent challenges that need to be addressed. Additionally, attention should be paid to gastrointestinal reactions, cardiac toxicity, kidney toxicity, and other adverse reactions during treatment. Arsenic is also often used to treat APL. Many studies have shown that all-trans retinoic acid (ATRA) combined with arsenic can achieve good results. In clinical practice, the combination of ATRA and arsenic can improve the overall effective rate of the treatment and reduce the recurrence rate.

In traditional Chinese medicine, arsenic-containing products include realgar (As_2_S_2_), orpiment (As_2_S_3_) and Pi-Shi (As_2_O_3_). Among them, the polypill of traditional Chinese medicine containing realgar is widely used. According to the 2020 edition of the Chinese Pharmacopoeia, 37 kinds of traditional Chinese patent medicines and simple preparations contain realgar. In *Shennong’s Herbal Classic of Materia Medica*, realgar is recorded as follows: ‘golden stone, pungent, and warm, can control cold and heat, rat fistula, sore, gangrene, and dead muscle, and kill white insect poison.’ Therefore, realgar has a history of over a thousand years as a medicinal herb in China. In the *Pharmacopoeia of the People’s Republic of China 2020* (Commission, 2020), it is recorded as a pungent, warm, and toxic agent that is beneficial to the liver and large intestine, and it has the effects of detoxifying and killing insects, eliminating dampness and phlegm, and treating malaria. It is mainly used for treating carbuncles, boils, snake and insect bites, abdominal pain, convulsions, and malaria. Orpiment is a monoclinic crystal system mineral mainly composed of arsenic trisulfide; it is highly toxic, mild in nature, and spicy in taste. It is beneficial to the liver and large intestine, and it has the effects of detoxification, insect disinfestation, dampness and phlegm elimination, and malaria treatment. It is mainly used to treat carbuncle and swelling, snake and insect bites, abdominal pain caused by parasites, convulsions, epilepsy, and malaria. In nature, realgar and orpiment often co-exist (Cao et al., 2011), so people call it Yuanyang Stone. Pi-Shi (derived from Arsenolite, Arsenopyrite, Realgar, or Orpiment) has a pungent taste, causes high fever, and exhibits high toxicity. It is beneficial for the lungs and liver, and it has the effects of removing carrion for external use and relieving asthma for internal use. The refined product obtained from the sublimation of Pi-Shi is arsenic trioxide. It is one of the oldest poisons, and it has been used as a drug for more than 2,400 years (Jiang et al., 2006). After entering the human body, it can destroy some cell respiratory enzymes (Yuan, 2010), making the tissue cells unable to obtain oxygen and die. It can also strongly stimulate the gastrointestinal mucosa, damage the liver, and, in serious cases, lead to death due to respiratory and circulatory failure. Chinese medicine prescriptions record that it can treat cold phlegm, asthma, malaria, dysentery, syphilis, hemorrhoids, scrofula, chancre, tinea, ulcers, and persistent carrion.

This article reviews the clinical application, pharmacological effects, and toxicity of arsenic to avoid its toxicity, enhance its therapeutic effect, and provide a theoretical basis for the clinical medication and development of arsenic agents.

## 2 Clinical applications of arsenic and its preparations

Arsenic is commonly used in clinical applications to treat hematological diseases, bone marrow, coronary heart disease, urogenital diseases, immune diseases, skin diseases or skin injuries, asthma, dental diseases, and tumors. Among them, the treatment of tumors and hematological diseases is its main application pathway, and the most documented one is for the treatment of APL. Among all forms of arsenic, ATO is the most commonly used.

### 2.1 Hematology

#### 2.1.1 Leukemia

APL is a unique subtype of acute myeloid leukemia (AML), accounting for approximately 10%–15% of AML cases. Amadori et al. (2005) reported that good tolerability has been found in clinical studies of arsenic trioxide, and its development reveals its widespread applicability in patients with hematological malignancies. Arsenic-containing traditional Chinese medicine, such as realgar, can be applied in treating APL in clinical practice. In the study by Guan et al. (2002), after 20 patients with APL (resistant to ATRA) were treated with realgar (3.0–3.75 g/day), the total effective rate (complete response (CR) + partial response (PR)) was 90%, and the time required to reach CR was 31–60 days. ATO has therapeutic effects on APL, involving the central nervous system. After multiple ineffective treatments, a 34-year-old patient with APL was treated with ATO monotherapy for 25 days. The patient’s neurological performance improved, bone marrow molecules were completely relieved, and mother cells in cerebrospinal fluid were significantly reduced, indicating the efficacy of ATO in treating APL recurrence involving the central nervous system (Zappasodi et al., 2011).

ATO or arsenious acid monotherapy has therapeutic effects on APL. Ascorbic acid (AA) can expand the treatment range of ATO and improve the clinical results of ATO monotherapy (Yedjou et al., 2008). Mathews et al. (2010) reported 72 patients with APL who were treated with a single dose of ATO, with a CR rate of 86.1%. The estimated 5-year event-free survival rate, disease-free survival (DFS) rate, and overall survival (OS) rate were 69%, 80%, and 74.2%, respectively. Ghavamzadeh et al., 2011) reported 197 patients with APL who were treated with a single dose of 0.15 mg/kg ATO, with a CR rate of 85.8% and a 5-year DFS of 66.7%. Shen et al. (1997) reported 10 cases of patients with APL, with a dose of 10 mg/day ATO. After treatment, the CR rate was 90%, and the required duration of ATO treatment to obtain CR was 28–44 days (median of 38 days). Jiang et al. (2008) reported 153 patients with APL, with a dose of 10 mg/day ATO. The CR rate was 80.21% (77/96) in the ATRA group and 92.98% (53/57) in the arsenite group.

Clinical studies have shown that ATRA combined with ATO is effective in the treatment of APL, and it is superior to using ATRA or ATO alone (Table 1).

**TABLE 1:** Statistics on the therapeutic effect of ATRA combined with ATO.

Patient (case)	Grouping (case)	Treatment	ATO dosage	CR (%)	Recurrence rate (%)	Three-year survival rate (%)	Efficacy rate (%)	Author
63	A:33	A: ATRA + ATO	10 mg/d	A: 60.61	-	-	A: 96.97	Wu (2020)
	B:30	B: ATRA		B: 63.33			B: 80.00	
298	A:177	A: ATRA + ATO	10 mg/d	-	A: 6.1	A: 92.9; B: 78.0 (no recurrence)	-	Lu et al. (2015)
	B:116	B: ATRA			B: 22.0			
		+ chemotherapy						
106	106	ATRA + ATO	10 mg/d	87.7	11.3	-	87.7	Gao et al. (2017)
40	40	ATRA + ATO	0.15 mg/kg	85	-	56 (18 months)	85	Soignet et al. (2001)
		+ chemotherapy						
36	36	ATRA + ATO	10 mg/d	94.4	-	-	94.4	Zhao et al. (2009)
50	A:25	A: ATRA + ATO	0.1%	A: 92	-	-	A: 92	Li (2020b)
	B:25	B: ATO		B: 68			B: 68	
90	D:47	D: ATRA/ATO	0.16 mg/kg	D: 89.36	-	-	D: 89.36	Li et al. (2021a)
	L:43	L: ATRA + ATO		L: 95.35			L: 95.35	
78	A:39	A: ATO	6 mg/m^2^	-	-	-	A: 66.67	Qiu et al. (2020)
	B:39	B: ATRA + ATO					B: 87.18	
82	82	ATRA + ATO	-	-	-	85	92	Ravandi et al. (2009)
481	A:244	A: ATRA + ATO + chemotherapy	-	-	-	A: 80; B: 63 (non-event)	-	Powell et al. (2010)
	B:237	B: ATRA				A: 90; B: 70 (No disease)		
		+ chemotherapy						
70	A:35	A: ATRA	10 mg/d	-	A: 51.43 (3 years)	A: 65.71	A: 54.29	Liu (2020c)
	B:35	+ chemotherapy			B: 11.43 (3 years)	B: 91.43	B: 82.86	
		B: ATRA + ATO + chemotherapy						
52	A:26	A: ATRA	2%	A: 23.1	-	-	A: 76.9	Shan (2020)
	B:26	B: ATRA + ATO		B: 38.5			B: 96.2	
23	A:12	A: ATRA + ATO	0.16 mg/kg	A: 91.7	-	-	A: 91.7	Lu et al. (2006)
	B:11	B: ATRA		B:72.7			B:72.7	
60	A:30	A: ATRA	0.16 mg/kg	A: 66.67	A: 40.00	-	A: 66.67	Zhang et al. (2018)
	B:30	+ chemotherapy		B: 90	B: 16.67		B: 90	
		B: ATRA + ATO + chemotherapy						
90	A:45	A: ATRA	10 mg/d	A: 82.22	-	-	A: 82.22	Wang (2020)
	B:45	B: ATRA + ATO		B: 95.56			B: 95.56	

Except for retinoic acid, ATO can also be used with other drugs to treat APL, such as HHT, IDA (Chen et al., 2018), and Fu Fang Qing Dai Pian (including realgar) (Qu et al., 2018).

The treatment of patients with recurrent APL via arsenic is also relatively safe and effective (Table 2).

**TABLE 2:** Statistics on arsenic in the clinical treatment of recurrent APL.

APL recurrent (case)	Dose of arsenic	Complete response rate	Author
20	As_2_O_3_ 10 mg/d (adult) and 0.16 mg/kg (children)	60% (12 cases)	Zhang (2017)
8	As_2_O_3_ 10 mg/d	100% (8 cases)	Kwong et al. (2001)
47	As_2_O_3_ 10 mg/d	85.1% (40 cases)	Niu et al., (1999)
40	As_2_O_3_ 0.15 mg/kg/d	85% (34 cases)	Soignet et al. (2001)
12	0.06–0.2 mg/kg ATO	91.67% (11 cases)	Soignet et al. (1998)
388	As_2_O_3_ 10 mg/d	74.13%	Ma et al. (2005)
15	As_2_O_3_ 10 mg/d	100%	Zhang et al. (2001)
41	Treatment group: arsenious acid 5 mg/d (2 children 2 mg/d–3 mg/d); control group: arsenious acid 10 mg/d	Treatment group: 80%	Guan et al. (2003)
		Control group: 81.9%	

However, the high cost of ATO treatment for APL is one of the reasons that hinder its clinical application (Zhu et al., 2016). Oral arsenic not only demonstrates clinical efficacy comparable with intravenous preparations but also demonstrates better safety, improved quality of life, and reduced patient medical costs, which promote its clinical application (Zhu et al., 2019). Zhu et al. (2014) reported that 20 non-high-risk APL volunteers were treated with oral arsenic (realgar and indigo) and ATRA, and they achieved CR after a median time of 29.5 days, indicating that the use of these two oral molecularly targeted drugs without chemotherapy is also effective, convenient, and economical. Ravandi et al. (2020) observed 12 patients with advanced hematological malignancies who received oral ORH-2014 (a novel oral ATO formulation), and their results showed that 15 mg of ORH-2014 is safe and bioavailable compared with an approved dose (0.15 mg/kg) of intravenous As_2_O_3_.

#### 2.1.2 Myelodysplastic syndrome

Myelodysplastic syndrome (MDS) is a clonal disease and a common hematological malignancy. It is a pathological or ineffective hematopoietic disease caused by the abnormal proliferation of bone marrow hematopoietic stem cells (Li et al., 2015a). ATO has a good effect on the treatment of MDS. The combination of ATO and thalidomide is a feasible treatment for MDS (Lengfelder et al., 2012), and ATO is an important drug that plays a major therapeutic role. For example, in 70 patients with MDS in the hematology department of Yangguang Ronghe Hospital (Zhu, 2020), thalidomide alone achieved a total effective rate of only 51.4%, whereas the combination of ATO and thalidomide achieved a total effective rate of 68.6%. In addition, the combination of azacitidine and ATO for the treatment of elderly MDS (Xie et al., 2022), which involves continuous treatment with azacitidine (75 mg/m^2^, subcutaneous injection) for 7 days and intravenous infusion of ATO (7 mg/m^2^, intravenous infusion), is more effective than using azacitidine alone, achieving a total effective rate of 76.92%, whereas azacitidine alone only achieves a total effective rate of 50.00%. Arsenic acid can inhibit myeloid clones, accelerate cell apoptosis (Emadi et al., 2010), and have significant clinical efficacy in treating MDS. The combination of ATO, cytarabine, aclacinomycin, and granulocyte colony-stimulating factor regimens can effectively treat medium-to-high-risk MDSs (Li, 2017), induce apoptosis of abnormal cloned cells, prolong patient survival, and reduce adverse reactions. Lu et al. (2019) used decitabine combined with ATO induction therapy to treat 39 patients with medium-to-high-risk MDS. Among them, 26 patients achieved clinical response, including 7 cases of CR.

Arsenic-containing traditional Chinese medicine is also a feasible path for treating MDS. Zhou et al. (2020) and others believed that arsenic-containing traditional Chinese medicine could treat MDS by regulating abnormal hypomethylation. The arsenic-containing compound Qinghuang powder is also a safe and effective method for treating MDS, as its daily dose can be adjusted without increasing clinical toxicity (Zhu et al., 2017).

#### 2.1.3 Multiple myeloma

Clinical research on arsenic compounds, especially ATO, has clinical activity in patients with recurrent/refractory multiple myeloma (MM) (Berenson et al., 2006). However, if ATO is used improperly (dosage or treatment method is inappropriate) in the treatment of MM, it can be poisonous (Rousselot et al., 2004).

Treating MM with ATO alone is an option in clinical practice. Hussein et al. (2004) reported the treatment of 24 MM patients with 0.25 mg/kg/d As_2_O_3_, with a response rate (RR) of 43%. The adverse reactions were mild, and patients can tolerate the treatment of As_2_O_3_ at this dose. Tian et al. (2006) observed the therapeutic effect of ATO on 14 patients with MM. The total effective rate was 64%, and the adverse reactions were relatively mild. Moreover, the combination of ATO and vitamin C has a certain therapeutic effect on MM (Bahlis et al., 2002; Li et al., 2015b).

### 2.2 Tumor (solid tumor)

#### 2.2.1 Liver cancer

Treating liver cancer with arsenic alone has certain clinical value. Zhu et al. (2003) reported 17 patients with liver cancer who were not suitable for surgery and underwent continuous regional chemotherapy with ATO (20 mg/day using a micropump). The results showed that six patients had a tumor volume reduction of more than 50% (PR = 35.2%), no new lesions appeared, eight patients had a reduction of 10%–49% (effective rate of 41.1%), one patient remained unchanged, and two patients had a tumor volume increase of more than 25%. Fu et al. (2006) observed 25 patients with advanced primary liver cancer who received a single daily dose of ATO injection (10 mg) via the intravenous drip for 10 days as a cycle; the treatment was repeated for 2 weeks intermittently. The results showed 4 cases of partial remission (PR), 17 cases of no change (NC), and 4 cases of progressive disease (PD), with a remission rate of 16.0%. The main adverse reactions were gastrointestinal reactions, bone marrow suppression, and liver function changes.

In addition to using arsenic alone for treatment, it can be combined with other drugs for treatment, such as 5-fluorouracil (Chen et al., 2008), sorafenib (Wu et al., 2019), and chemotherapy drugs (Liu et al., 2006).

The combination of arsenic trioxide/arsenite and transcatheter arterial chemoembolization (TACE) has a certain therapeutic effect on liver cancer (Table 3).

**TABLE 3:** Statistics on the therapeutic effect of arsenic trioxide/arsenite combined with TACE.

Cases of liver cancer	Grouping and treatment method	Objective response rate	Survival rate	Author
62	Control group: TACE	15.6%	46.0% (1 year)	Yan et al. (2013)
	As_2_O_3_ group: As_2_O_3_ + TACE	20.0%	52.0% (1 year)	
55	Control group: TACE	31.0%	51.7% (1 year)	Cui et al. (2006)
	Treatment group: TACE + As_2_O_3_ (20 mg/d)	34.6%	80.8% (1 year)	
124	Control group: TACE	58.06%	83.87% (half a year), 45.16% (1 year), and 17.74% (2 years)	Xie et al. (2017)
	Treatment group: TACE + As_2_O_3_ (10 mg/d)	70.97%	88.70% (half a year), 62.90% (1 year), and 27.42% (2 years)	
64	TACE group	43.3%。	60.0% (1 year), 44.4% (2 years), and 25.0% (3 years)	Qi et al. (2003)
	TACE + As_2_O_3_ group	73.5%	88.2% (1 year), 63.3% (2 years), and 42.1% (3 years)	
82	Control group: TACE	53.66%	60.98% (1 year) and 34.15% (2 years)	Bian et al. (2021)
	Treatment group: As_2_O_3_ (20 mg) + TACE	75.61%	82.93% (1 year) and 46.34% (2 years)	
86	Control group: TACE	8.9%	-	Zhou et al. (2007b)
	As_2_O_3_ group: TACE + As_2_O_3_(20 mg)	14.6%	-	
125	A group: TACE + As_2_O_3_(10 mg/d)	81.96%	98.4% (half a year), 93.4% (1 year), and 75.9% (2 years)	Wang et al. (2015)
	B group: TACE	59.37%	87.5% (half a year), 64.1% (1 year), and 50.9% (2 years)	
80	Treatment group: As_2_O_3_(10 mg) + TACE + Fuzheng Sanjie Decoction	37.5%	75% (1 year)	Wan et al. (2021)
	Control group: As_2_O_3_(10 mg) + TACE	30%	55% (1 year)	
30	TACE + arsenious oxide	77%	-	Tian et al. (2006)
60	Control group: As_2_O_3_ + TACE	20.00%	-	Wang et al. (2016)
	Treatment group: Ganxi tablets + As_2_O_3_ + TACE	26.67%	-	

#### 2.2.2 Lymphoma

When ATO is applied to lymphoma, intravenous injection of 0.25 mg/m^2^ combined with AA can lead to disease progression, but dosage changes can have therapeutic effects. Ravandi et al. (2003) reported a 29-year-old female patient with refractory Burkitt-like lymphoma who had a high lactate dehydrogenase (LDH) value. As_2_O_3_ (intravenous injection of 0.25 mg/m^2^ per day for 2 h) and AA (oral administration of 500 mg twice a day) were administered, followed by prednisone treatment. As a result of the side effects and further development of the disease, treatment was discontinued. However, due to its ability to reduce serum LDH, attempts were made to change the dosage of As_2_O_3_ to obtain a safe and effective treatment. Michel et al. (2003) applied As_2_O_3_ to two patients with cutaneous T-cell lymphoma, and the results showed that the first patient achieved PR, whereas the second patient remained stable. Huang et al. (2007) subsequently observed 23 patients with relapsed and refractory malignant lymphoma who were treated with As_2_O_3_. The As_2_O_3_ injection was slowly administered intravenously at a dose of 10 mg/m^2^. Among the 21 evaluable cases, they reported 3 cases with complete remission (CR) (14.3%), 10 cases with PR (47.6%), 5 cases with stable disease (SD) (23.8%), and 3 cases with PDs (14.3%). The response rate (RR) was 61.9%, indicating that As_2_O_3_ has a good therapeutic effect on relapsed and refractory malignant lymphoma and is worthy of further clinical trials.

### 2.3 Clinical pharmacokinetics of arsenic

When ATO is used to treat APL, its content in serum and urine gradually decreases, but it is also sufficient to treat APL. Ghiuzeli et al. (2022) observed the metabolism of ATO in 25 patients with APL and measured inorganic As (iAs), monomethylarsenic acid (MMA), and dimethylarsenic acid (DMA). After the administration of ATO, the total plasma iAs increased, followed by a rapid decrease, reaching a low level within 4–6 h. Fukai et al. (2006) studied Japanese patients with relapsed APL who received daily treatment with 0.08 mg/kg ATO for 35 days to achieve CR. Serum and urine samples were collected on the fourth and fifth days of ATO consolidation treatment, and the test results showed that arsenic in the serum was sufficient to exert therapeutic effects on APL cells. Guo et al. (2021) suggested that infusion methods and combination therapy may affect arsenic metabolism. In 305 patients with APL treated with ATO, the distribution trend of arsenic in plasma was as follows: DMAV > AsIII > MMAV > AsV during continuous slow infusion and DMAV > MMAV > AsIII > AsV during routine infusion.

Arsenic-containing traditional Chinese medicine has non-active ingredients that cannot be absorbed, and the degree of absorption of arsenic in different proportions of compound arsenic also varies. The main components HgS and As_2_S_2_ of cinnabar and realgar are inactive ingredients that cannot be absorbed by the body (Ye et al., 2005). Liu et al. (2016) studied the proportion relationship between indigo naturalis and realgar compound, and they found that an increase in the proportion of indigo naturalis can promote the absorption of arsenic in rats.

## 3 Pharmacological effects of arsenic

Among all the pharmacological effects of arsenic, anti-tumor is the most important and dose-dependent, with a dosage range of 0.01–80 μmol/L, and the concentration of arsenic does not exceed 20 μmol/L in most studies.

### 3.1 Anti-tumor (solid tumor)

Cancer is one of the most common public health issues worldwide (Zhang et al., 2019a). Arsenic and its preparations can inhibit tumor growth by regulating inflammatory signaling pathways, and they are used for the treatment of various solid tumors. ATO can inhibit the growth of many different types of cancer cells and promote apoptosis (Murgo, 2001).

#### 3.1.1 Lung cancer

Lung cancer is the main cause of cancer death (Sung et al., 2021). Smoking or exposure to second-hand smoke, chronic obstructive pulmonary disease, occupational exposure (asbestos, cadmium, silicon, diesel waste, nickel, coal smoke, and coal smoke ash), history of radon exposure, and family history of first-degree relatives with lung cancer are related to the occurrence of lung cancer (Malhotra et al., 2016; Turner et al., 2020; Fan et al., 2022). Arsenic-containing traditional Chinese medicine orpiment is a monoclinic ore that is mainly composed of arsenic trisulfide and is highly toxic. Liu et al. (2008) conducted pharmacological and toxicological studies on orpiment, and they reported that it can effectively treat certain malignant tumors and external skin diseases.

*In vitro*, arsenic compounds have been shown to have cytotoxic effects, induce tumor cell apoptosis, inhibit tumor cells, and affect tumor angiogenesis in a dose-dependent manner. Dong et al. (2007) found that after treating human lung cancer cell lines with high concentrations of As_2_O_3_, the proliferation inhibition rate of cancer cells can reach over 80%. Chen et al. (2020) used As_2_O_3_ (0, 2.5, 5, 10, 15, 20, and 25 μmol/mL) to intervene in the human large-cell lung cancer NCI-H460 cell line, verifying that ATO may inhibit proliferation, block the cell cycle, and induce the apoptosis of NCI-H460 cells by downregulating the expression of Bcl-2, CDK1, and c-Myc proteins; upregulating Bax protein expression; and activating caspase-9 and caspase-3 proteases. Liang et al. (2020) treated lung adenocarcinoma A549 cells with different concentrations of ATO (1, 3, and 6 μmol/L) for 12, 24, and 48 h, indicating that ATO can induce apoptosis in lung adenocarcinoma A549 cells by downregulating the level of cell cycle genes, upregulating the content of apoptosis-related factors, and reducing MAPK/ERK signaling. In animal experiments, ATO can also inhibit tumor growth. Chen et al. (2021a) treated 5- to 6-week-old BALB/c-nu male nude mice with As_2_O_3_ (2.5 and 5.0 mg/kg) and implanted 125 I particles to construct a lung cancer transplant tumor model. The results showed that both the As_2_O_3_ (2.5 and 5.0 mg/kg) treatment group and the combination treatment group could significantly inhibit tumor growth in mice, indicating that As_2_O_3_ and its combination with 125 I particle implantation could inhibit tumor growth in lung cancer transplant tumor mice.

#### 3.1.2 Liver cancer

Liver cancer is one of the most common malignant tumors in clinical practice and has the highest mortality rate. Among numerous treatment options, ATO has always played an important role as a broad-spectrum anti-cancer drug (Wu et al., 2006). In recent years, domestic and international research has confirmed its positive role in inhibiting the growth of solid tumor cells and promoting cell apoptosis (Zhao et al., 2015a; Shen et al., 2017). Given that the liver is a detoxifying organ, the local concentration of ATO is high, which is conducive to its anti-liver cancer effect. Its anti-liver cancer effect is mainly achieved by inducing the apoptosis of liver cancer cells (Hu et al., 2003).

Arsenic can inhibit tumor cell proliferation and induce cell apoptosis. *In vitro*, ATO can act on multiple targets and induce apoptosis in liver cancer cells, and ATO only inhibits liver cancer cells without affecting normal liver cells (Zhao, 2016). Gao et al. (2010) demonstrated that As_2_O_3_ can activate the JNKs/AP-1 cell apoptosis pathway by inducing growth inhibition and DNA damage-induced protein 45α (GADD45α) expression in the human liver cancer cell line HepG2. However, under the same arsenic exposure conditions, GADD45α was not induced to be expressed in normal human liver cells HL7702 and LO2.

In the liver cancer transplant tumor model, administration of ATO can promote tumor cell apoptosis (Chen et al., 2000; Li et al., 2001) and inhibit tumor growth (Wang et al., 2020a; Duan et al., 2021).

#### 3.1.3 Reproductive system tumors

Breast cancer is a malignant tumor with the highest incidence rate among women. In developed countries such as Europe and the United States, breast cancer accounts for 25%–30% of female malignant tumors (Siegel et al., 2019). Liu et al. (2020b) treated the human breast cancer cell line MCF7 with arsenic trioxide to reduce the release of adenosine diphosphate (ADP) from platelets, thereby inhibiting platelet aggregation induced by MCF7. Triple-negative breast cancer mostly shows invasive growth with a high degree of malignancy. Research on its treatment still lacks breakthrough progress (Cserni et al., 2021). The expression of promyelocytic leukemia (PML) protein in breast cancer tissue is significantly higher than that in adjacent tissue and normal breast tissue, and it has potential anti-cancer ability (Gamell et al., 2014). Zhang et al. (2021a) proved that As_2_O_3_ can enhance the inhibitory effect of PML on the proliferation, invasion, and migration of triple-negative breast cancer cells to treat triple-negative breast cancer. Ji et al. (2021) treated the human triple-negative breast cancer cell line MDA-MB-231 with 2, 4, 6, 8, 10, and 12 μmol/L As_2_O_3_; with the increase in concentration, they found that ATO can downregulate the expression of macrophage colony-stimulating factor 1 (CSF1) and inhibit the chemotaxis of the triple-negative breast cancer cell line MDA-MB-231 to human monocyte line U937.

ATO can promote apoptosis of breast cancer cells (Chen et al., 2003), inhibit the growth of breast cancer (Kozono et al., 2018), and have a cytotoxic effect on breast cancer cells (Karasulu et al., 2004). Liao et al. (2009) has shown that abnormalities in the Hedgehog pathway are closely related to the occurrence and development of some tumors. The combination of arsenic disulfide and itraconazole has a synergistic effect, jointly downregulating the expression of SMO and Glil proteins in the Hedgehog signaling pathway to achieve anti-tumor effects (Wang et al., 2021b).

ATO can be used as an alternative chemotherapy for ovarian cancer, inducing apoptosis of ovarian cancer cells (Uslu et al., 2000), inhibiting drug resistance in tumor cells (Du et al., 2001), and directly acting on tumor cells. In addition, As_2_O_3_ can induce cell apoptosis by inhibiting the activity of glutathione peroxidase (GPX) and increasing the production of H_2_O_2_ in mitochondria (Dai et al., 1999). As_2_O_3_ can inhibit microtubule depolymerization and regulate key regulatory proteins of cell cycle G2-M phase transformation so that ovarian cancer, cervical cancer, and breast cancer cells can stagnate in the G2/M phase and then induce apoptosis (Ling et al., 2002). For ovarian cancer drug-resistant cell lines, As_2_O_3_ can still play an inhibitory role. Huang et al. (2002) showed that the human ovarian cancer-resistant cell line 3AO/cDDP has cisplatin resistance. As_2_O_3_ has a significant inhibitory effect on the growth of human ovarian cancer cell lines 3AO and 3AO/cDDP, and the inhibitory effect of As_2_O_3_ on the growth of 3AO/cDDP cells is not significant compared with 3AO cells, so As_2_O_3_ is equally effective on 3AO/cDDP cells as it is on 3AO cells.

#### 3.1.4 Lymphoma

Lymphoma is divided into Hodgkin’s lymphoma and non-Hodgkin’s lymphoma (Katabi et al., 2017). Some arsenic compounds, such as arsenic trioxide, realgar, and sodium arsenate, have significant effects in treating lymphoma. As_2_O_3_ can inhibit the proliferation of DLBCL and Jurkat lymphoma cells in a concentration- and time-dependent manner (Wang et al., 2013). Lo et al. (2014) found that As_2_O_3_ induces apoptosis in MCL lymphocytes in a time- and dose-dependent manner while inhibiting cyclin D1. Gao et al. (2009) found that As_2_O_3_ at eight different concentrations (0.1, 0.3, 0.5, 0.6, 0.8, 1.0, 2.0, and 3.0 mol/L) has an inhibitory effect on EL4 cell proliferation. Yoon et al. (2016) implanted lymphoma Daudi cells subcutaneously into nude mice; after 14 days, the mice were injected with 3.5 and 7.0 mg/kg sodium arsenate. The results showed that the tumor volume in the treatment group was smaller than that in the control group, indicating that sodium arsenate can inhibit the growth of lymphoma cells. Wang et al. (2012) found that the combination of As_2_O_3_ and ethacrynic acid can induce apoptosis in lymphoma cells. The two synergistically activate the c-Jun-NH_2_-terminal kinase and reduce the expression of Mcl-1 protein, leading to a decrease in mitochondrial transmembrane potential and the release of cytochrome c. Subsequently, caspase-3 and caspase-9 are activated, inducing apoptosis in the cells.

### 3.2 Influence on the blood system

#### 3.2.1 Influence on leukemia (hematological tumors)

ATO, whose mechanism in treating leukemia is related to inducing cell differentiation and apoptosis, is a multi-target and multi-pathway anti-tumor drug (Wu et al., 2017). In leukemia, ATO is most commonly used in the treatment of APL, and it is mainly used in AML. AML is one of the most common hematological malignancies in clinical practice (Xiao et al., 2020a). ATO can inhibit survival and induce apoptosis on AML cells, and STAT3 inhibitor Stattic can enhance this effect (Wu et al., 2021a). The mechanism may be related to the increase in ROS caused by the inhibition of Nrf2 and Nrf2 downstream gene expression (Nrf2 enhances the drug resistance of AML cells (Sekeres et al., 2012)). The synergistic application of ATO with decitabine (Gore et al., 2010; Lo-Coco et al., 2015) or rapamycin (Xiao et al., 2020a) can inhibit AML cells. Low concentrations of ATO combined with NK cells can inhibit the proliferation of the AML KG1a cell line (Wang et al., 2021a).

APL is a special subtype of AML characterized by the infinite proliferation of tumor cells in the body’s hematopoietic system (Abedin et al., 2016), which inhibits the production of normal cells (Zheng, 2018). It has unique cytogenetic changes. ATO has a dose-dependent dual effect on APL cells, triggering cell apoptosis at high doses and inducing partial cell differentiation at low doses (Zhou et al., 2007a; Zheng et al., 2008; Chen et al., 2021b). In addition, ATO can also be rapidly converted into trivalent methylation metabolites in the liver through methyltransferase (AS3MT) and redistributed to the blood of patients with APL receiving ATO treatment (Maimaitiyiming et al., 2020). The combination of ATO with tanshinone IIA (Wang et al., 2020b) or Puerariae Radix Flavones (Chen et al., 2021c) can induce apoptosis of promyelocytes. The adverse prognostic factors for treating APL may include additional gene mutations, including FLT3-ITD (Shen et al., 2015). ATRA combined with ATO can improve the prognosis of patients with APL by inhibiting the FLT3-ITD signaling pathway (Wang et al., 2017). In addition, the synergistic treatment of ATRA and arsenic significantly weakens the inhibitory effect of FLT3-ITD on ATRA, restores the degradation of PML-RARα induced by ATRA, promotes recombination of PML nucleosomes, activates P53, and eradicates APL (Esnault et al., 2019).

#### 3.2.2 Multiple myeloma

ATO can induce apoptosis in MM cells and inhibit their growth and proliferation. Mathas et al. (2003) used *in vitro* cultured myeloma cells to show that ATO can inhibit the activity of NF-κB induced by tumor necrosis factor α and prevent NF-κB from upregulating IL-6 and other genes, thereby reducing IL-6 concentration, eliminating IL-6-mediated proliferation of MM cells, and weakening their ability to resist apoptosis. However, when Rousselot et al. (2004) treated 10 patients with advanced MM with arsenic trioxide, they found that ATO as a single agent does not produce any significant response. When used in combination with melarsoprol, ATO can inhibit the activity and growth of plasma cells in the bone marrow or blood of patients with MM, induce apoptosis, and ultimately lead to myeloma cell death (Estrov et al., 1999). Bahlis et al. (2002) demonstrated that As_2_O_3_ inhibits growth and induces apoptosis in drug-resistant MM cells *in vitro*. The addition of vitamin C can enhance the activity of As_2_O_3_. The combination of ATO and vitamin C has a good therapeutic effect on patients with MM (Fu et al., 2020). It can downregulate the expression of matrix metalloproteinase MMP-13 [involved in the occurrence and development of MM (Lin et al., 2016)] and nuclear factor NF-κB-p65 protein and upregulate the expression of CCAAT enhancer-binding protein C/EBPα, with a good prognosis. Arsenic acid can increase the production of superoxide and inhibit the redox system, leading to a large accumulation of superoxide in the body, directly damaging mitochondria, and inducing cell apoptosis through the internal mitochondrial apoptosis pathway (Sánchez et al., 2008). It can also reduce the secretion of IL-6 by preventing MM cells from adhering to bone marrow stromal cells, slow down the progression of MM, and weaken the anti-apoptotic effect mediated by IL-6 (Ravandi, 2004). Hu et al. (2021a) confirmed that ATO can inhibit the proliferation and promote the apoptosis of MM cells by inhibiting the activation of the PI3K/AKT pathway.

### 3.3 Antivirus

Arsenic has anti-viral effects in the form of ATO or As_2_S_2_. Low concentrations of ATO can effectively inhibit the replication of hepatitis C virus in sub-gene replicon cells carrying hepatitis C virus, and ATO affects interferon against hepatitis C virus, making it a potential anti-hepatitis C virus drug (Hwang et al., 2004). The main component of realgar is As_2_S_2_, with a dosage of 100–200 mg each time. As_2_S_2_ has slight toxicity to the human body, but it has a strong inactivation effect on viral proteases (Ding, 2012), thereby playing an anti-viral role. Moreover, realgar has an inactivating effect on various proteases in human hosts, such as DNA-linking enzymes, DNA primer enzymes, DNA polymerase, and RNA reverse transcriptase, making it a full-spectrum anti-viral drug (Ren, 2004).

## 4 Toxicity of arsenic and its preparations

Arsenic is widely found in soil, rock, and water (Li et al., 2016) and may contaminate drinking water, air, food, and industrial products. Among various arsenic exposure pathways, arsenic poisoning occurs mainly through drinking arsenic-contaminated water and coal combustion (Zhang et al., 2020a). Arsenic poisoning is a serious threat to human health, causing skin toxicity, blood toxicity, eye toxicity, and genito-urinary toxicity, of which carcinogenic toxicity is the most common. Statistical data (P. et al., 2013) showed that China is gradually becoming one of the countries with the greatest impact and highest incidence of endemic arsenicosis. Costa (2019) concluded that knockdown of H3.3 alone induces carcinogenesis, and the replacement of functional H3.3 proteins by increased H3.1 proteins may be one of the mechanisms of arsenic carcinogenesis.

### 4.1 Pathways of arsenic poisoning

Arsenic is susceptible to environmental contamination, causing arsenic poisoning in humans after contaminating food, water, air, and soil (Hughes et al., 2011). Environmental contamination by arsenic remains a major public health problem in many countries worldwide (Bolt, 2012; Bolt, 2013b), the most important of which are drinking water contamination (groundwater contamination) (Rahaman et al., 2021), atmospheric arsenic contamination, coal combustion-type contamination, environmental contamination from mining activities, and waste deposits (Bolt, 2013a).

Of all the modes of arsenic exposure, infection by ingestion of contaminated groundwater is most prevalent (Caussy, 2003) (more than 200 million people are estimated to be at risk of arsenic exposure (Bundschuh et al., 2013)). In recent years, the main causes of serious groundwater contamination include industrial wastewater and pesticide and fertilizer discharges that have led to arsenic entering natural water bodies (Shakoor et al., 2017). In China, 19 provinces have groundwater arsenic concentrations exceeding 10 μg/L; in some areas, more than 50% of the water has arsenic concentrations exceeding 50 μg/L, with a maximum of 2,330 μg/L (Jia et al., 2018). Arsenic contamination of groundwater is also prevalent in many countries, such as Bangladesh, Central Europe, Chile, Mexico, and the United States, where the arsenic in the water exceeds the WHO guidance value of 10 μg/L. As early as 2001, groundwater wells in Vietnam had arsenic levels exceeding 3,000 μg/L (Christen, 2001).

Arsenic can cause cardiovascular disease (CVD), skin cancer, respiratory cancer, and other diseases after it is ingested or inhaled in the atmosphere (Welch et al., 1982; Engel et al., 1994; Yu et al., 2006). The European Union (EU) has a limit of 6 ng/m^3^ for atmospheric arsenic. Zhang et al. (2020b) found that air arsenic concentrations in 2005 were highest in Chile and eastern China, with mean values of 8.34 and 5.63 ng/m^3^, respectively. By 2015, as a result of the rapid growth of the Indian coal quantity, India’s average atmospheric concentrations of arsenic (4.57 ng/m^3^) were higher than those of eastern China (4.38 ng/m^3^). The combined effect of arsenic in the atmosphere and groundwater may significantly increase the risk of cancer. Coal burning and copper smelting are the main sources of arsenic in atmospheric PM2.5 (Jiang et al., 2015), which can cause serious air pollution (Liu et al., 2020a). He et al. (2019) detected the arsenic content in PM2.5 in Baoding, China, and found that the indoor and outdoor concentrations were 3.7–36.8 and 9.6–59.5 ng/m^3^, respectively. The average arsenic concentration exceeded 6 ng/m^3^, indicating serious atmospheric arsenic pollution.

### 4.2 Dermal toxicity

The WHO defines arsenicosis as a chronic health disorder that, if sustained intake of arsenic in excess of a safe dose for more than 6 months, patients usually present with skin lesions characterized by melanosis, keratosis pilaris (Tian et al., 2020), palmoplantar keratosis (Patel et al., 2021), dermatitis, hyper- and hypopigmentation of the skin (Chandra et al., 2022), verrucous hyperplasia, skin cancers, and thickening of the skin (Liu et al., 2001); in severe cases, even squamous cell carcinomas and basal cell carcinomas may develop (Zhou, Q. et al., 2018). Das et al. (2008) concluded that skin damage is generally the initial symptom of arsenic poisoning and is the basis for diagnosis. Therefore, in Taiwan, skin pigmentation and hyperkeratotic skin damage are common indicators of arsenic exposure (Mead, 2005). Although skin lesions can occur in any part of the body, they are more common in non-exposed areas such as the trunk, buttocks, and thighs and appear as raindrops or extensive florid black or tan spots (Yang, 2007).

Long-term use of herbs containing large amounts of arsenic can lead to chronic arsenic poisoning, and skin lesions are the most common and earliest manifestation in patients with arsenic poisoning. The skin manifestations of patients with arsenic poisoning are detailed in Table 4.

**TABLE 4:** Statistics on skin manifestations in patients with arsenic poisoning.

Year	Patient	Cause of poisoning dermal	Manifestation of toxicity	Author
1955	A case of syphilis in a 48-year-old male	Neoarsphenamine treatment	Rashes all over the body with itching, followed by higher fever and denser rash	Qian et al. (1965a)
1950–1965	20 patients with arsenic dermatitis	Arsenic causes dermatitis	Reactions such as Herxheimer’s reaction, day 9 erythema, warning signs, and generalized exfoliative dermatitis occurred	Qian et al. (1965b)
1974	74 patients with acute, subacute, and chronic poisoning	Taking certain brands of herbal preparations from Singapore (containing inorganic arsenic compounds)	The majority (92%) showed skin manifestations	Tay (1974)
1999	A woman with acute arsenic poisoning	Ingestion of 8 ± 16 g sodium arsenite	Erythroderma with vesicles and pustules	Bartolomé et al. (1999)
2019	A 73-year-old male of Cantonese origin	Daily consumption of homemade traditional medicine “herbal balls” (including arsenic) for 5 years	The patient developed hyperkeratosis on the soles of the feet and palms of the hands, scattered actinic keratosis pilaris lesions on the dorsal and plantar surfaces of the hands, and freckles on the torso	Spilchuk et al. (2019)
2020	A male patient with acute promyelocytic leukemia	ATO therapy	Severe skin rash	Zheng et al. (2021)
2020	A 60-year-old man from Guangdong	Irregular use of homemade traditional digestive herbs (contains more than 80 kinds)	He presented with skin discoloration on the trunk for 5 years and raised lesions on the palm for 3 years. Finally, he died from chronic arsenic poisoning	Teng (2020)
2021	202 cases of arsenism caused by coal-burning	Arsenic poisoning caused by burning coal in Yuzhang of Qianxinan prefecture, Guizhou province	All patients had different degrees of skin damage, and 84 of them had severe skin damage	Liu et al. (2021)

A close relationship exists between the concentration of arsenic exposure and skin damage, with the degree of skin damage gradually increasing as the concentration of arsenic exposure increases (Guo et al., 2010; Li et al., 2021a).

Most cases of arsenic-induced skin cancer are caused by poisoning from the administration of arsenic-containing drugs. The induction of skin cancer by arsenic mixtures used for medicinal purposes was reported as early as 1888 (Furst, 1984). Minkowitz (1964) reported a case of arsenic poisoning from the administration of Fowler’s solution, which caused a variety of characteristic keratoconus and neoplastic lesions, including primary cutaneous epidermoid carcinoma and papillary epidermoid carcinoma of the left posterior region of the laryngeal ring. Siefring et al. (2018) reported a case of 46-year-old Vietnamese men who developed extensive, multiple, and concurrent cutaneous squamous cell carcinoma in non-sun-exposed skin areas after taking arsenic-based traditional medicine for chronic plaque psoriasis. Additionally, arsenic-induced skin cancer starts from basal germinal cells (Chang et al., 1998; Matsui et al., 1999).

### 4.3 Genito-urinary toxicity

Arsenic can cause damage to the reproductive system; according to statistics, China has more than 3 million people with high arsenic exposure (Zhang, 2015). Long-term exposure to arsenic has certain health hazards (Zhang et al., 2021b). Research suggests that arsenic compounds are a class of environmental estrogens (EEs) (Stoica et al., 2000), which can affect the human endocrine system and nervous system development and interfere with reproductive function.

#### 4.3.1 Effects on germ cells

Studies have found that arsenic above a certain dose has serious effects on male sexual function, such as reduction in testicular weight and alterations in steroidogenesis and spermatogenesis (Machado-Neves, 2022). Arsenic causes a decrease in sperm count (Zhang et al., 2003; Sanghamitra et al., 2008; Zeng et al., 2019) and sperm deformities (Shalat et al., 1996; Zhang et al., 1996; Gao et al., 2019) in rats and mice. Arsenic can also interfere with spermatogenesis by inhibiting the Ddx3y gene (Luo et al., 2020). Other studies (Dai et al., 2020) found that arsenic may indirectly alter spermatogenesis and development by affecting the expression of VEGF and VEGFR2, testicular mesenchymal cells, and blood vessels, which in turn may collectively affect male fertility.

#### 4.3.2 Effects on embryos and offspring

During development, chronic exposure to arsenic and its compounds can easily cross the placenta and accumulate in the embryo’s brain (Qu et al., 2020), enter the fetus through the blood–placenta barrier, and affect the embryo’s development, leading to congenital malformations. In severe cases, miscarriage and stillbirth can occur (Kang, 2004). Thus, arsenic has embryotoxicity. Robinson et al. (2011) showed that As has a dose-dependent effect on gene expression in mouse embryos and has potential embryotoxic effects. Mirkes et al. (1992) found that acute *in vitro* exposure of embryos to sodium arsenite (50 μmol/L) on day 10 of gestation in rats was embryotoxic, as evidenced by decreased growth and abnormal development. Rodríguez et al. (2002) showed that inorganic arsenic has embryotoxic and teratogenic effects on experimental animals. Li et al. (2009) exposed zebrafish embryos to high concentrations (0.5–10 mmol/L) of arsenite pollutants, and they detected reduced survival of embryos in the arsenic-treated group and abnormalities such as delayed hatching, growth retardation, and morphological changes. Colomina et al. (1997) administered 10 mg/kg sodium arsenite via gavage to mice on days 15–18 of gestation and observed significant delays in ear opening and eye-opening times in the offspring of mice exposed to sodium arsenite after natural delivery. Chen et al. (2023) found inverse associations between prenatal arsenic exposure and the neurodevelopment of children at 2 years old, even at low exposure levels.

#### 4.3.3 Effects on estrogen receptors

ATO has an effect on the estrogen receptor (ER). Chen et al. (2002) showed through *in vitro* tests with breast cancer cells that high doses of As_2_O_3_ reduce cell survival, whereas low doses inhibit ERα gene expression, which is restored 24 h after the removal of arsenide. Da Silva et al. (2017) found that mice injected subcutaneously with ATO for 5 consecutive weeks had downregulated levels of AR expression in mesenchymal and epididymal epithelial cells. Rosenblatt et al. (2009) found that ATO downregulates AR expression levels and inhibits AR transcriptional activity in prostate cell lines (PC3, LNCap, and LAPC4 cells).

#### 4.3.4 Effects on the urinary system

Arsenic that enters the human body is mainly excreted in the urine, which inevitably affects the urinary system. Ghosh et al. (2008) have shown that humans, rhesus monkeys, rabbits, rats, hamsters, mice, dogs, and cows excrete MMA and DMA in their urine; studies in Taiwan, Bangladesh, and China found that low levels of DMA cause MMA to accumulate in the body, and elevated concentrations of MMA increase the risk of arsenic exposure-related diseases such as bladder cancer (Tseng et al., 2005; Abhyankar et al., 2012; Lin et al., 2018).

Arsenic exposure causes bladder cancer and produces significant renal pathological changes. Müller et al. (2007) studied the epidemiology and tumor toxicology of collectives exposed to arsenic orally, and they found that uroepithelial and renal cancers may be a consequence of chronic (oral) arsenic ingestion. Fukushima et al. (2005) found that organoarsenic dicarboxylic acid is carcinogenic to the bladder of rats in the multi-organ carcinogenesis bioassay model (DMBDD model) and carcinogenicity test. A controlled study (Ferreccio et al., 1998) in Chile found an association between bladder cancer and arsenic exposure. In an epidemiological survey of 42 villages near staghorn mines in the United States, Lamm et al. (2005) found that residents with arsenic levels in drinking water above 400 μg·L^−1^ have a significantly increased risk of bladder cancer. Arsenic enters the organism, causing renal injury and renal dysfunction. Zhong et al. (2018) found that sub-acute arsenic poisoning causes a decline in renal function by studying cases of patients with sub-acute arsenic poisoning in Ganzhou People’s Hospital.

**FIGURE 1:**
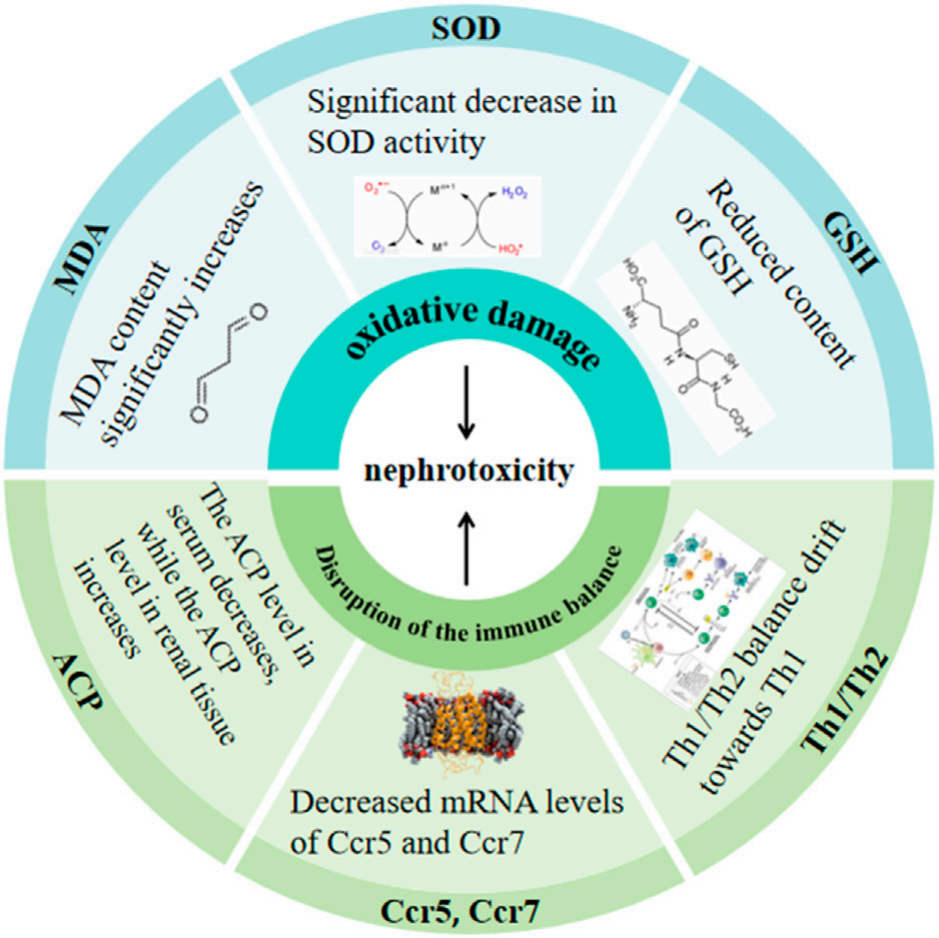
Arsenic-induced nephrotoxicity.

Arsenic can induce nephrotoxicity through oxidative damage and disruption of the immune balance within the kidney. Details are presented in Figure 1 (Li et al., 1997; Sun et al., 1998; Li et al., 2020a; Xu et al., 2020; Li et al., 2021b).

### 4.4 Neurotoxicity

Arsenic is neurotoxic, and long-term arsenic exposure can be observed in the central nervous system’s inhibitory symptoms, including headache, drowsiness, irritability, memory loss, convulsions, coma, and peripheral neuritis accompanied by muscle weakness (Kang, 2004). The conditions of severe cases can lead to death (Smith et al., 2000). Moreover, when the peripheral nervous system is impaired, peripheral neuritis and vegetative nerve disorders are common signs and symptoms (Zhao et al., 2010). Arsenic-induced neurotoxicity consists of oxidative stress, apoptosis, thiamine deficiency, and decreased acetylcholinesterase activity (Mochizuki, 2019). Arsenic-induced neuropathy can occur in acute and chronic poisoning, but it is more common in acute arsenic poisoning. Acute arsenic poisoning often presents with peripheral neuropathy within a few weeks (usually 1–3 weeks) after poisoning (You, 2000), and Ramírez-Campos et al. (1998) reported a case of peripheral neuropathy in only 3 days. Kuang (2004) reported a case of paralysis of respiratory muscles in a patient with sub-acute arsenic poisoning 15 days after poisoning.

#### 4.4.1 Toxicity to the central nervous system

The toxicity of arsenic to the central nervous system is caused by multiple mechanisms (Garza-Lombó et al., 2019). For example, it can cause damage such as oxidative damage (Chattopadhyay et al., 2002; Perveen et al., 2017; Pan et al., 2018; Zhou et al., 2018) to brain tissue, disruption of neurotransmitter metabolizing enzymes in brain tissue (Zhang et al., 2015), disruption of the cholinergic system (Tyler et al., 2014), and pathological changes in hippocampal tissue in the brain (Qin et al., 2017).

Arsenic exposure can damage hippocampal tissues. A previous study reported (Zhao et al., 2013) that arsenic can cross the blood–brain barrier, thereby damaging the basal ganglia, hippocampus, and cerebral cortex. In a study by Zhang et al. (2020c), in a rat model of arsenic poisoning induced by NaAsO_2_, the hippocampal tissues of rats in the model group developed severe pathological lesions, such as neuronal degeneration, hemorrhage in part of the hippocampal tissues, disorganization of the structural arrangement of the hippocampal tissues, and glioblast hyperplasia. Yang et al. (2021a) proved that the metabolites of realgar in rats are iAs, MMA, and DMA; MMA and DMA can accumulate in brain tissue through the blood–brain barrier, which can cause a decrease in the ability to recognize new things, resulting in the damage of hippocampal neuronal cells. Sheng et al. (2021) used the mouse hippocampal neuronal cell line HT-22 as a subject and found that arsenic can affect the expression of ERK, JNK, p38, PI3K/Akt, and Nrf2/HO-1 proteins and promote the apoptosis of hippocampal neurons.

Arsenic affects intelligence and reduces learning memory in mice and rats. Calderón et al. (2001) found a significant decrease in children’s verbal intelligence quotient (IQ) with increasing levels of urinary arsenic, and children with high levels of urinary arsenic have lower prolonged memory scores and verbal abstraction scores than children with low levels of urinary arsenic. Wasserman et al. (2004) and Wasserman et al. (2007) found a negative correlation between reduced intelligence and drinking water arsenic levels (water arsenic concentrations of 117.8 ± 145.2 and 120.1 ± 134.4 μg/L, respectively) in 10- and 6-year-old children from arsenic-exposed drinking water in Araihazar district, Bangladesh. Zhang et al. (2019b) found that arsenic exposure leads to a decrease in learning and memory ability in mice, and Sun et al. (2017) reported that chronic arsenic exposure results in a significant decrease in learning and memory ability, accompanied by an upregulation of the expression of GRP78, PERK, ATF4, and CHOP proteins in the hippocampal cells of rats. Krüger et al. (2006) found that sodium arsenite significantly inhibits LTP action in hippocampal brain slices of adult rats (two to four months) and young rats (14–21 days), thereby inhibiting learning and memory. Luo et al. (2009) showed that drinking water exposure to arsenic (2.72, 13.6, and 68 mg/L) for 3 months affects spatial memory, hippocampal ultrastructure, and expression of n-methyl-d-aspartate receptor genes in rats.

#### 4.4.2 Effects on the peripheral nervous system

Arsenic toxicity to the peripheral nervous system has been reported in many clinical settings. For example, Xu et al. (2008) reported that 104 workers overdosed on arsenic after a pipeline leak at a copper smelting plant; at 17 days after admission to the hospital, many of the subjects (45, 43.3%) developed peripheral neuropathy, with 25 of them (24.0%) experiencing decreased motor and sensory nerve conduction velocities. Valappil et al. (2019) reported a 9-year-old girl with sub-acute sensory-motor peripheral neuropathy for 2 weeks, starting 3 weeks after taking a locally produced conventional drug for obesity; the patient had high serum arsenic levels, and nerve conduction on admission showed axonal sensory motor neuropathy with slow conduction. Gherardi et al. (1990) reported a young woman with sleeping sickness who was treated with melarsoprol (an organic arsenic compound); On the 38th day after administration of the drug, she had massive distal Wöhler degeneration of the peripheral nerves and abnormalities of the dorsal ganglion and spinal cord. Xu (2018) divided 186 cases of patients with PNOCAP (a peripheral neuropathy caused by long-term exposure of patients to arsenic) into two groups according to the level of arsenide concentrations in the patient’s body, namely, the low-arsenic group (the value of arsenic <30 μg/g) and the high-arsenic group (the value of arsenic >30 μg/g); 146 patients (78.49%) showed the phenomenon of numbness of the limbs, and the motor nerve conduction velocity of the high-arsenic group was lower than that of the low-arsenic group. Arsenic affects peripheral nerve mechanical nociception in rats. Lai et al. (2020) administered arsenic (25 and 100 mg·L^−1^ sodium arsenite) to rats via drinking water; at the 20th week of poisoning, compared with the control group, the mean mechanical pain thresholds of the two groups decreased by 38% and 54%, respectively, and the expression levels of myelin basic protein of nerve fibers and the inflammatory factors IFN-γ and TNF-α were elevated.

### 4.5 Hepatotoxicity

The liver, as a metabolic organ, is the main site of As_2_O_3_ metabolism, and retention of high concentrations during its metabolism is highly likely to cause liver injury (Acosta et al., 1985). After acute or chronic arsenic exposure, toxicities such as inflammation, genotoxicity, mutagenicity, and carcinogenic effects may occur (Mandal et al., 2002), among which arsenic damage to liver cells is one of the most serious types of arsenic toxicity. Arsenic is often used in the treatment of APL, and hepatic adverse effects have been observed during treatment. For example, Mathews et al. (2006) reported that As_2_O_3_ alone causes hepatotoxic reactions when used in the treatment of patients with newly diagnosed APL, with a 38.2% incidence of hepatotoxicity in 76 patients. Hao et al. (2013) showed that approximately 24.4% of APL patients treated with As_2_O_3_ develop liver injury, most of which is mild and moderate, and APL is mainly characterized by elevated alanine aminotransferase (ALT), alanine transaminase (AST), and gamma-glutamyl transpeptidase (GGT). Grade 3–4 liver injury was also observed in 25%–63% of patients with APL treated with As_2_O_3_ in combination with ATTRA by Burnett et al. (2015). A previous study reported (Nishikawa et al., 2002) that the oral administration of DMA has a promotional effect on hepatocellular carcinoma (HCC) in rats, causing a significant increase in the level of hepatic 8-oxodG and inducing an increase in P-450 synthesis. In addition, Miao et al. (2011) found that 600 mg·kg^−1^ realgar (arsenic-containing traditional Chinese medicines) can cause partial liver cell edema in mice.

The potential mechanisms of arsenic-induced hepatotoxicity include oxidative damage, promotion of pro-inflammatory factor expression, induction of hepatocyte apoptosis, and liver fibrosis (Renu et al., 2021). Arsenic-induced oxidative damage is associated with ROS formation in a time-dependent manner (Santra et al., 2022). The mechanism of arsenic-induced apoptosis may be related to the activation of p38, which increases the level of p-p38 protein expression (Hu et al., 2020a).

**FIGURE 2:**
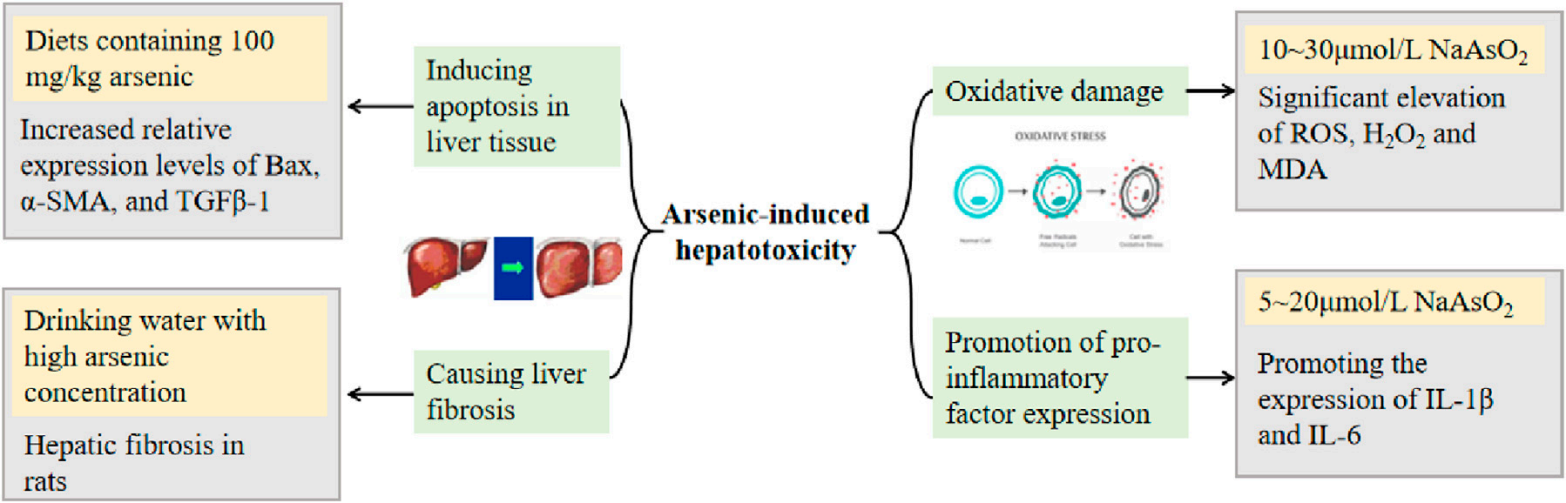
Arsenic-induced hepatotoxicity.

Many studies on the mechanism of arsenic toxicity have been reported through animal experiments. Chi et al. (2019) found that arsenic interferes with gene expression, including the p53 signaling pathway, upregulates many oncogenes highly expressed in HCC, and downregulates a number of oncogenes, suggesting an increased risk for the development of HCC after injection of arsenious acid into routinely reared normal mice and mice in which antibiotics were used to disrupt intestinal flora. Li et al. (2015b) induced hepatic injury in mice by gavage administration of As_2_O_3_; mice showed a decrease in total antioxidant capacity (T-AOC) and glutathione (GSH) levels, an increase in MDA content, a significant increase in serum ALT and AST, and a significant elevation of hepatocyte cellular structure and morphological changes in liver histopathological tests compared with those of blank group mice. Liu et al. (2018) showed that As_2_O_3_ causes a significant increase in the percentage of apoptotic cells in the liver of male Hy-line chickens. In addition, arsenic affects the metabolism of Ca and Mg in the liver, causing liver damage in rats and affecting the ability to repair the damage. Specific concentrations and toxicity mechanisms are detailed in Figure 2 (Yan et al., 2009; Hu et al., 2021b; Deng et al., 2021; Zeng et al., 2022; Zhao et al., 2022).

### 4.6 Circulatory system toxicity

CVD poses a serious risk to human health; increased mortality from CVD is associated with exposure to high levels of arsenic, and As exposure is associated with atherosclerosis (Wang et al., 2002; Wang et al., 2010). The molecular mechanism of arsenic-induced cardiovascular toxicity is not fully understood, but epigenetic changes, increased platelet aggregation, and increased oxidative stress are all related to it (Kononenko et al., 2021).

Arsenic can directly damage the capillaries and act on the vasoconstrictor center, paralyzing the smooth muscle of the vascular wall, increasing permeability, reducing blood volume, and aggravating organ damage. The interaction of arsenic with arachidonic acid biotransformation (Bunderson et al., 2004), affecting vasoconstriction and diastole, may be one of the mechanisms responsible for cardiovascular toxicity. Through a review of the literature, Stea et al. (2014) discovered an association between low-level arsenic exposure and CVD and its complications. Ochoa-Martínez Á et al. (2019) evaluated prognostic CVD biomarkers in Mexican women exposed to iAs in drinking water and found that the most sensitive CVD biomarkers are plasma atherosclerotic index (AIP), plasma asymmetric dimethylarginine (ADMA), and adipocyte fatty acid-binding protein (FABP4), which are associated with arsenic exposure.

**FIGURE 3:**
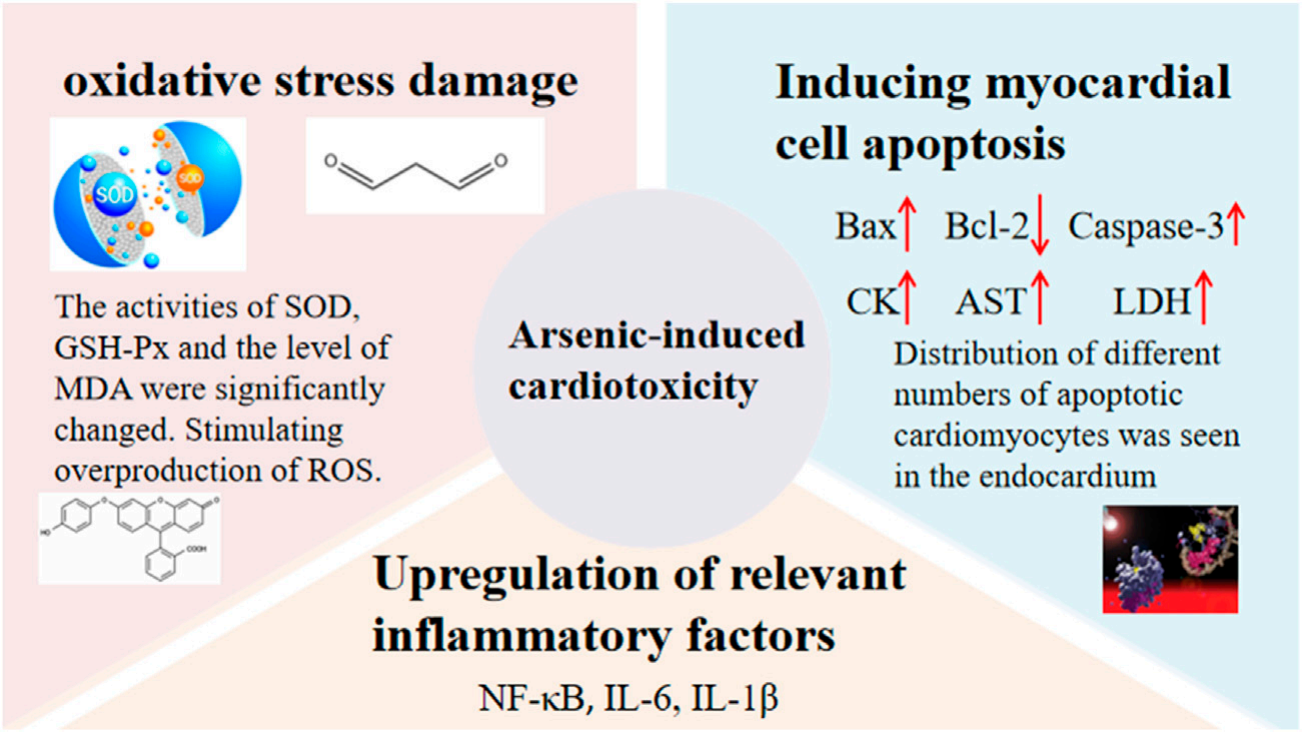
Arsenic-induced cardiotoxicity.

Cardiotoxicity has also been found to be significant when ATO is used therapeutically (Zhang, F. et al., 2018), and cardiotoxicity is mainly of two types: (sub)acute cardiotoxicity and chronic cardiotoxicity. ATO has multiple mechanisms of inducing cardiotoxicity, such as oxidative stress damage, upregulation of relevant inflammatory factors, and induction of myocardial damage (Haybar et al., 2019; Vineetha et al., 2019). Details are shown in Figure 3 (Saad et al., 2006; Han et al., 2010; Zhao et al., 2015b; Li et al., 2020c; Yang et al., 2021b; Zhao et al., 2021b; Yang et al., 2021c; Xu et al., 2021).

In addition, arsenic exposure causes anemia, leukopenia, lymphocytosis, and thrombocytopenia (Li et al., 2000; Islam et al., 2004; Lee et al., 2004; Wang et al., 2009a; Mahmud et al., 2009; Parvez et al., 2017; Sun et al., 2019).

### 4.7 Effects on the endocrine system

Experimental evidence by Tseng et al. (2002) demonstrated that chronic long-term exposure to arsenic can induce the development of diabetes (Rahaman et al., 2021), especially type 2 diabetes. Overall, urinary arsenic levels may be higher in the type 2 diabetic population than in the general population, and urinary arsenic levels are associated with glycosylated hemoglobin levels in the type 2 diabetic population (Feseke et al., 2015). Lai et al. (1994) detected excessive arsenic exposure in drinking well water in a highly endemic area of Taiwan with crow’s foot disease, and the prevalence of diabetes mellitus in these villages was higher than that of the general population of Taiwan by a factor of approximately two; their research group was the first to determine a relationship between arsenic exposure and diabetes mellitus. By adopting a case-series approach, Xiao et al. (2020b) found that the average urinary arsenic level in the type 2 diabetic population was 38.81 μg/L, which was higher than the average urinary arsenic level in the general population of 13.72 μg/L (Tian et al., 2015). Through clinical observation, Hu et al. (2020b) found that low arsenic levels in maternal blood may promote the onset and development of maternal gestational diabetes mellitus (GDM). Navas-Acien et al. (2006) found that arsenic may affect the development of diabetes through other mechanisms such as oxidative stress, inflammation, or apoptosis; all non-specific mechanisms have been implicated in the development of type 2 diabetes. A study involving 45 pre-diabetic and 65 diabetic patients in New York City (Wu et al., 2021b) demonstrated that high MMA (MMA%) and low DMA (DMA%), i.e., lower ability to methylate arsenic, in urine are associated with poor glycemic control and diabetes. In addition, arsenic causes an increase in blood glucose levels and disorders of glucose metabolism in the body (Wang et al., 2009b; Xie et al., 2020; Dai et al., 2021).

### 4.8 Respiratory toxicity

Chronic arsenic exposure can cause lung cancer (Ng et al., 2003). The mechanism of arsenic carcinogenesis may be related to ERs, oxidative stress, high levels of EGFR proteins, and disruption of this balance between pro- and anti-apoptotic factors.

Arsenic is considered an endocrine disruptor with estrogen-like effects, capable of interfering with the expression of ER; the abnormal expression of ER is closely related to the development of lung cancer (He et al., 2015). Cheng et al. (2019) persistently stained the human bronchial epithelial cell line 16-HBE with 2.5 μM of sodium arsenite. The results indicated that sodium arsenite induced the malignant transformation of the human bronchial epithelial cell line 16-HBE, and ERs may be involved in arsenic-induced malignant transformation of cells. Arsenic may induce lung carcinogenesis through oxidative stress (Zhao et al., 2021a) and can cause cells to proliferate abnormally and eventually become malignant by upsetting this balance between pro- and anti-apoptotic factors (Zhu et al., 2020; Chen et al., 2021c).

Environmental arsenic exposure or misuse of arsenic-containing medications can lead to cancers of the respiratory system. Buechley (1963) found that lung cancer mortality is directly proportional to the amount of arsenic in the environment. Half of the miners in Sachsen died of lung cancer (Hueper, 1956). A meta-analysis of five articles using a fixed-effects model was conducted by Chen et al. (2014). The results of their analysis showed that the exposed group was approximately 1.37 times more likely to develop lung cancer relative to the non-exposed group. Thus, arsenic exposure may cause a high incidence of lung cancer and may be a high-risk factor for the development of lung cancer. Mabuchi et al. (1979) documented a case of lung cancer due to exposure to inorganic arsenic pesticides. Robson et al. (1963) reported a 45-year-old woman who had been taking arsenic-containing Fowler’s solution for intermittent bullous pemphigoid for more than 15 years, and an examination showed undifferentiated carcinoma in the right main bronchus. Lee et al. (2005) reported a case of a 53-year-old lifelong non-smoker with chronic asthma who was treated with arsenic-containing herbal medicines for 10 years as a child, and the patient was diagnosed with Bowen’s disease and extensive stage small cell carcinoma of lung (SCLC) at 10 and 47 years after arsenic exposure, respectively.

### 4.9 Other toxicity

Arsenic’s effects on chromosomes have been extensively studied, with either trivalent or pentavalent arsenic causing chromosomal aberrations and increasing sister chromatid monosome exchange (An et al., 1999; Nuta et al., 2021). Arsenic has been tested *in vitro* and *in vivo* for genotoxicity (Patlolla et al., 2012; Zheng et al., 2020; Qian et al., 2021). Genotoxicity and proteotoxicity, which arsenic can cause, may be interrelated and together contribute to proteinopathies (Wysocki et al., 2023).

Arsenic is also toxic to the immune system (Giles et al., 2022). Arsenic accumulation inhibits lymphocyte proliferation, migration, and activation; induces T-cell apoptosis; and suppresses T-cell activity by increasing oxidative stress and decreasing IL-2 secretion (Huang et al., 2019).

In addition, arsenic increases pressure within the pulp cavity, destroying capillaries and causing bleeding (Luo et al., 2004).

## 5 Discussion

In clinical practice, ATO is usually administered intravenously at a dose of 10 mg/day for the treatment of hematological diseases, including leukemia, MDS, and MM. When ATO is used in combination with ATRA in the treatment of APL, it can achieve good therapeutic effects. In addition, it is often used for the treatment of solid tumors, such as liver cancer and lymphoma cancer. Among arsenic-containing traditional Chinese medicine, realgar is widely used in clinical practice and can be used to treat APL, MDS, and lymphoma. However, clinical pharmacokinetic research is relatively scarce, and further research is needed.

In terms of pharmacology, arsenic is most commonly used for anti-tumor functions because it can inhibit the growth of solid tumor cells, promote cell apoptosis, resist tumor angiogenesis, promote partial differentiation, degrade certain specific fusion proteins, downregulate the expression of the Bcl-2 protein, downregulate mitochondrial transmembrane potential ∆Ψm, and activate cysteine protease caspases. In addition to its anti-tumor effects, arsenic can fight against viruses and asthma. However, research on the pharmacokinetics of arsenic remains limited. The pharmacological effects of arsenic are dose-dependent, ranging from 0.01 μmol/L to 80 μmol/L, with arsenic concentrations not exceeding 20 μmol/L in most studies.

Arsenic not only has anti-tumor effects but also has carcinogenic and teratogenic toxicity. It can cause environmental pollution, such as coal burning, arsenic poisoning, and drinking water poisoning. Skin damage, such as rash or dermatitis, is usually the primary manifestation and sign of arsenic poisoning. In severe cases, it can evolve into skin cancer, and dosage plays a crucial role. The pathways of arsenic poisoning mainly include ingestion of contaminated groundwater, arsenic pollution in the atmosphere, and coal-fired pollution. For example, Waalkes et al. (2003) demonstrated that C3H mice exposed to high levels of inorganic arsenic (42.5 and 85 ppm) can develop HCC, lung cancer, liver tumors, and ovarian tumors. The important mechanisms of arsenic carcinogenesis are methylation metabolism and oxidative damage. The toxicity of arsenic is also dose-dependent. Within the effective concentration range of arsenic (less than 20 μmol/L), it can cause toxic damage. For example, 5 μmol/L sodium arsenite can induce liver oxidative damage and promote the expression of pro-inflammatory factors; 10 and 20 μmol/L sodium arsenite can promote the proliferation of human lung cancer A549 cells, and 15 μmol/L sodium arsenite can induce myocardial damage. When the concentration is high, it is likely to cause toxic damage. For example, sodium arsenite is embryotoxic to rats at 50 μmol/L; when the concentration of arsenic reaches 600 μmol/L, it causes obvious toxic damage to astrocytes (ACs) in the brain (Feng et al., 2019). Therefore, when applying it, a concentration of 20 μmol/L should not be exceeded, and the treatment concentration can be adjusted according to the patient’s physical condition.

It is necessary for the government to regularly monitor the concentration of arsenic. First, the government should control the concentration of arsenic in groundwater to not exceed 10 μg/L to prevent arsenic poisoning caused by ingestion of groundwater, as well as to prevent contamination of crops such as rice. For high-arsenic water exceeding this concentration, low-arsenic water can be mixed to achieve an acceptable arsenic concentration or used for laundry and other purposes. Second, the government should also limit the amount of arsenic in the atmosphere of residential areas to below 3 ng/m^3^, mainly by reducing coal pollution (implementing mandatory arsenic removal treatment for high arsenic coal). Third, the management of arsenic-containing pesticides and pharmaceuticals must be strengthened. For example, the packaging of arsenic-containing pesticides must be dyed red to prevent confusion with food. Arsenic-containing drugs, such as realgar, should be tested for arsenic content to control drug standards. For the general public, it is necessary to understand the dangers of high arsenic exposure and the sources of arsenic exposure, including crops, distinguish the use of low-arsenic water from high-arsenic water, and avoid ingesting high-arsenic water. In addition, people should also be aware of the signs of arsenic poisoning in order to seek timely medical attention.

## 6 Conclusion

As is well known, arsenic is a toxic metalloid element and a global pollutant. In the past 30 years, most studies and reports on arsenic research have focused on toxicity in various aspects, with relatively less research on pharmacology and clinical applications. Exposure to arsenic can cause various types of toxicity, including circulatory system toxicity, urogenital system toxicity, endocrine system toxicity, skin toxicity, neurotoxicity, hepatotoxicity, and respiratory system toxicity. Initially, arsenic exposure is mainly manifested as skin toxicity and causes significant toxicity to the liver, kidney, and nervous system. The main mechanisms by which arsenic induces liver and kidney toxicity are oxidative damage, disruption of immune balance in the kidneys, promotion of pro-inflammatory factor expression, induction of liver cell apoptosis, and initiation of liver fibrosis. The limited clinical application of arsenic is mainly due to its high toxicity and numerous adverse reactions, making it difficult to find a suitable dosage. Additionally, the dosage should be tailored to the individual’s physical condition. In previous studies, 10 mg/d was a commonly used dose for arsenic trioxide, and all-trans retinoic acid was a commonly used combination drug. However, there is still an urgent need for more research on arsenic in clinical pharmacokinetics to ensure the safety of clinical applications. The pharmacological effects of arsenic are dose-dependent and have been extensively studied in anti-tumor studies, with inhibitory effects on various solid tumors. However, there is still limited research on the pharmacokinetics of arsenic.

Therefore, attention should be paid to environmental arsenic pollution, prevention of arsenic poisoning, and risk assessment of arsenic. In addition, attention should also be paid to the relationship among dose, time, pharmacological effects, and toxicity. According to the theory of traditional Chinese medicine, future research should focus on combining medications to enhance efficacy and reduce toxicity.
